# Personalization of injection protocols to the individual patient’s blood volume and automated tube voltage selection (ATVS) in coronary CTA

**DOI:** 10.1371/journal.pone.0203682

**Published:** 2018-09-26

**Authors:** Nienke G. Eijsvoogel, Babs M. F. Hendriks, Jef L. Willigers, Bibi Martens, Luc F. Carati, Barbora Horehledova, Bastiaan L. J. H. Kietselaer, Harry J. G. M. Crijns, Joachim E. Wildberger, Marco Das

**Affiliations:** 1 Department of Radiology and Nuclear Medicine, Maastricht University Medical Centre, Maastricht, The Netherlands; 2 CARIM School for Cardiovascular Diseases, Maastricht University Medical Centre, Maastricht, The Netherlands; 3 Department of Cardiology, Maastricht University Medical Centre, Maastricht, The Netherlands; 4 Department of Diagnostic and Interventional Radiology, Helios Kliniken Duisburg, Duisburg, Germany; Nagoya University, JAPAN

## Abstract

**Purpose:**

The aim was to assess personalised contrast media (CM) protocols—based on patient’s blood volume (BV) and automated tube voltage selection (ATVS)—in coronary computed tomography angiography (CCTA).

**Methods:**

A total of 114 consecutive patients received an ECG-triggered or ECG-gated helical scan on a 3^rd^-generation dual-source CT with 70-120kV (ATVS) and 330mAs_qual.ref_. CM was adapted to BV, scan time (s) and kV. Image quality (IQ) was assessed in a 17-segment coronary model using attenuation values (HU), contrast-to-noise (CNR), signal-to-noise ratio (SNR) (objective IQ) and a Likert scale (subjective IQ: 1 = poor/2 = sufficient/3 = good/4 = excellent). ig

**Results:**

Patient distribution was: n = 60 for 70kV, n = 37 80kV and n = 17 90kV. Mean BV was 5.4±0.6L for men and 4.1±0.6L for women. Mean CM volume (300 mg I/mL) and flow rate were: 30.9±6.4mL and 3.3±0.5mL/s (70kV); 40.8±7.1mL and 4.5±0.6mL/s (80kV); 53.6±8.6mL and 5.7±0.6mL/s (90kV). Overall mean HU was >300HU in 98.2% (112/114) of patients. Overall mean attenuation was below 300HU in two scans (70kV) due to late scan timing. Of 1.661 segments, 95.4% was assessable. Mean CNR was 14±4(70kV), 13±3(80kV) and 14±4(90kV); mean SNR was 10±2(both 70kV+80kV) and 9±2(90kV). Objective IQ was comparable between kV settings, protocols and sex. Subjective IQ was diagnostic in all scans and excellent-sufficient in 95.4% of segments.

**Conclusions:**

Personalisation of CCTA CM injection protocols to BV and ATVS is a promising technique to tailor CM administration to the individual patient, while maintaining diagnostic IQ.

## Introduction

Cardiac diseases are the leading cause of death in the western world [1]. Growing evidence shows that cardiac diseases, especially coronary artery disease (CAD), worsen the prognosis in a broad variety of non-cardiac pathologies (e.g. in patients with chronic obstructive pulmonary disease or diabetes mellitus) [2–4]. Coronary computed tomography angiography (CCTA) is one of the non-invasive imaging techniques allowing imaging of the heart and coronary arteries. The high sensitivity (up to 95%) and negative predictive value (NPV; up to 99%) of CCTA make it a valuable tool in the assessment and ruling out of CAD, especially in patients with low to intermediate risk for CAD [5–9].

The relation between computed tomographic angiography (CTA) and the development of contrast-induced nephropathy (CIN) has recently been a topic of discussion [10]. Patients with advanced age and cardiac diseases are more prone to develop CIN. Therefore, it would be beneficial to adapt and individualise CM injection protocols to a level where the patient only receives the minimum amount of CM required, in combination with an appropriate iodine delivery rate (IDR), to produce a CCTA with diagnostic image quality (sufficient intravascular attenuation and contrast-to-noise (CNR)). Different techniques have been used to adjust CM injection protocols and no consensus exists on the optimal method. Previous studies have shown that adaptation of CM injection protocols to both tube voltage and body weight (BW) is feasible and results in lower CM volumes [11–14]. Another possibility is adjustment of CM injection protocols (flow rate and CM volume) to the patient’s estimated blood volume (BV). It is known that fatty tissue receives less BV compared to muscle tissue [15]. Thus, since the body habitus (fatty/muscle tissue) of men and women differ, the estimated BV between men and women of the same BW varies as well. Using BV, the sex of the patient is taken into account, which could be the next step in individualised diagnostic imaging.

To our knowledge, the feasibility of using BV in this context has not previously been investigated. Therefore, the aim of our study is to assess the diagnostic image quality when adjusting CM injection protocols to estimated BV of the individual patient and automated tube voltage selection (ATVS).

## Materials and methods

### Study design

The personalised injection protocol was incorporated in standard clinical care for patients receiving CCTA. The local ethical committee and institutional review board approved the study design and a waiver of written informed consent was obtained (MEC Academisch ziekenhuis Maastricht/Universiteit Maastricht; number 16-4-162).

All patients who underwent CCTA between July and October 2016 were eligible for inclusion. Inclusion criteria were: patients over 18 years old and referral for a contrast enhanced CCTA. Exclusion criteria: patients with previous severe adverse reaction to CM (hypotensive shock, respiratory arrest, cardiac arrest and/or convulsions [16]), age < 18 years, pregnancy or renal insufficiency (estimated glomerular filtration rate < 30 mL/min/1.73 m^2^).

Patient characteristics (age, height, BW, Body Mass Index (BMI), BV, heart rate (HR; bpm) and sex) and scan indications were prospectively collected and analysed.

### CT scan protocol

Where necessary, patients were prepared with beta blockade (oral or intravenous). All patients received sublingual nitro-glycerine (Nitrolingual pumpspray 0.4 mg, Isordil®, Pohl-Boskamp, Hohenlockstedt, Germany). All scans were performed on a 3^rd^-generation dual-source CT scanner (Somatom Force, Siemens Healthineers, Forchheim, Germany). Patients received either a prospectively ECG-triggered (‘high-pitch’ or ‘adaptive sequence’ [AS]) CCTA depending on the HR of the patient (HR ≤ 70 bpm high pitch protocol, HR 70–90 bpm AS protocol). The scans were performed with tube voltage settings set by automated tube voltage selection (ATVS, CAREkV^TM^, Siemens) with 100 kV_ref.qual_, slider position 11 and 330 mAs_ref.qual_ (CareDose 4D^TM^, Siemens). For patients with a pacemaker, a tube voltage of 120 kV was chosen by the technician to avoid artefacts caused by the leads. Slice collimation of 2*192*0.6 mm (high pitch) or 2*160*0.6 mm (AS) was used, 0.25 s rotation time and pitch value 3.2 (high pitch). Test bolus technique was used to determine scan delay. Because of the shorter scan times, especially with the prospective ‘high pitch’ protocol, the chance of missing the bolus increases. When the bolus tracking technique would be used, there is a chance the peak attenuation would be missed. Therefore, the test bolus technique was chosen. Image reconstruction was performed using 0.6 mm slice thickness and an (overlapping) increment of 0.4 mm. A Bv40 kernel (strength 2) was used for high pitch and Bv36 (strength 3) for AS (Advanced Modelled Iterative Reconstruction (ADMIRE), Siemens). Dose monitoring software (Radimetrics Enterprise Platform^TM^; Bayer Healthcare, Berlin, Germany) was used to record all dose-related parameters. This software automatically calculates the effective (organ) radiation dose (mSv), with help of Monte Carlo simulations, according to the latest guidelines of the International Commission on Radiological Protection (ICRP; ICRP 103).

### CM injection protocol

Iopromide (300 mg I/mL, Bayer Healthcare) was pre-warmed (37 ˚C) and administered using a dual-head CT power injector (Stellant, Bayer) via an 18–22 gauge intravenous injection catheter in either the left or right antecubital vein. All patients received an injection protocol based on their BV. First, a formula was developed to calculate the hypothetical concentration of contrast media in the blood of the patient (c; mg I/mL):

$$c=\frac{F*I_{c}}{CO}(1-e^{-\frac{CO}{V}*t})$$

This formula accounts for the flow rate of the CM injected (F; mL/s), the concentration of the CM used (I_c_; mg I/mL), the cardiac output (CO; mL/s), the volume of CM that remains in the heart (V; mL), the time of injection (t; s) and the patient factor for patient variability (e). This formula was then adapted to achieve an intravascular attenuation of ± 300 Hounsfield units (HU) in the coronary arteries. The known relation of intravascular attenuation (HU) and concentration of CM in the blood (HU = k*c; with k being a constant) [15, 17] and inverse relation of HU and tube voltage (HU = 1/kV) [11] was used to predict the expected intravascular attenuation (with a reference tube voltage of 120 kV):

$${HU}^{kVp}=k*\frac{F*I_{c}}{kVp}*patient\;factor$$

In this formula BV, calculated using the Nadler formula [18], was chosen as patient factor. The Nadler formula calculates the patient’s BV based on BW and height. The Nadler formula was inserted in the formula above. To help modify the formula for the individual patient, a ‘reference patient’ was inserted in the Nadler formula (height 1.80 m, BW 80 kg, tube voltage 120 kV, flow rate 7.0 mL/s and IDR 2.1 gI/s). The resulting intravascular attenuation of 350 HU was inserted as HU^kvp^ to ensure safe margins for reaching the diagnostic attenuation. The formula was then rewritten to calculate the flow rate (F) for each individual patient. The modified formula calculates the required flow rate for the individual patient and adjusts for BV and kV setting. With BV = (0.3669*h^3^) + (0.03219*w) + 0.6041 for men and BV = (0.3561*h^3^)+ (0.03308*w) + 0.1833 for women (w = BW in kg and h = height in cm):

F=0.0002*BV*kVp

**Fig 1:**
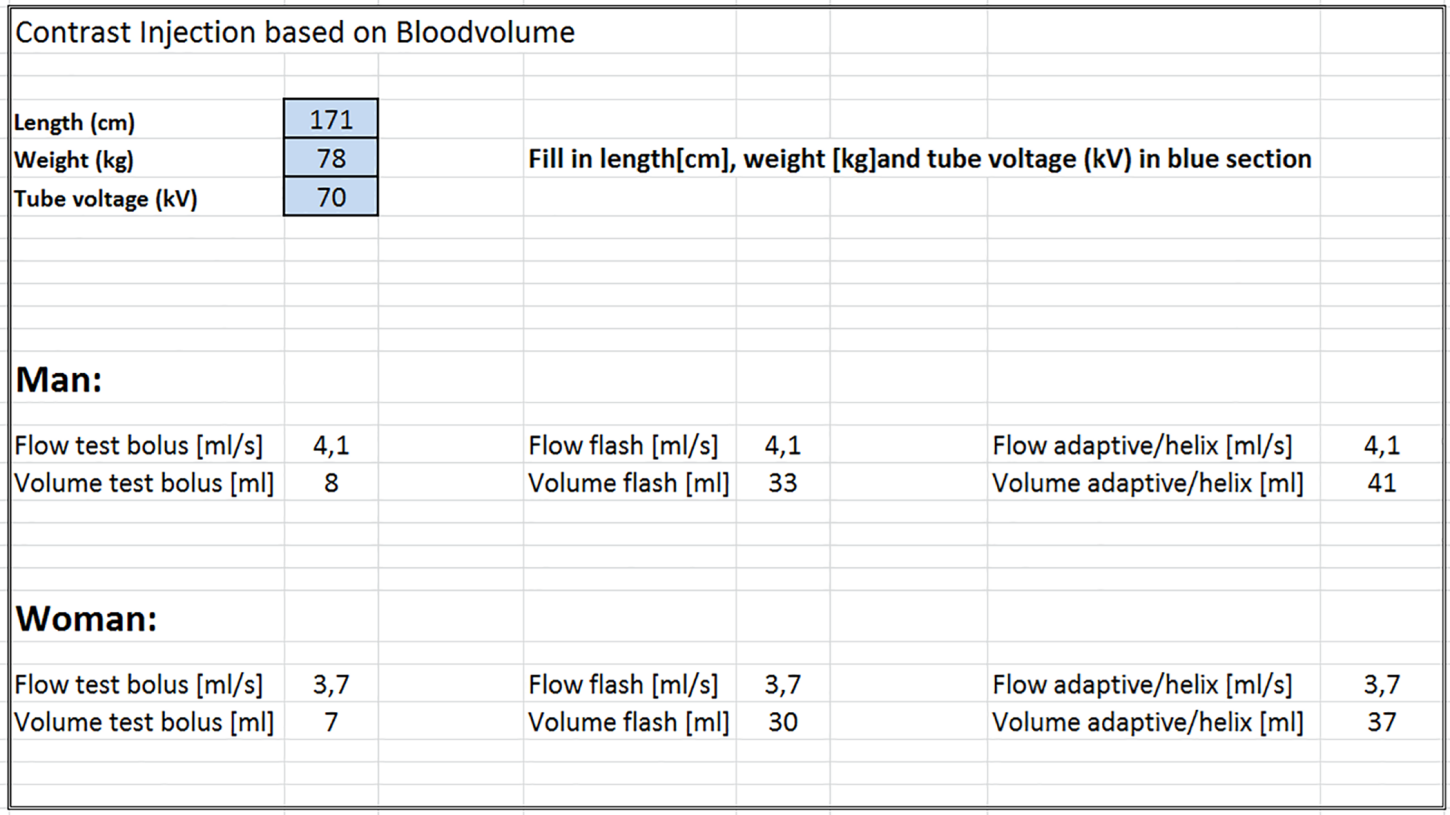
Example of excel file used to calculate the individual patient’s injection protocol. Patient characteristics where inserted in the blue cells, the excel file calculated the needed injection protocol.

The CM volume was then determined with the calculated flow rate (mL/s) and the known scan time (s) of the scan protocols (8 s for high pitch protocol and 10 s for AS protocol). An Excel file was assembled with the formulas to make sure the technicians only had to choose the right protocol and change the patient parameters accordingly (**Fig 1**).

All relevant CM injection parameters were recorded by CM monitoring software (Certegra^TM^ Informatics Solution, Bayer). The total iodine load (TIL) injection duration (ID) and IDR were additionally calculated.

### Data analysis

Dedicated software (Syngo.via^TM^ VB10B_HF04, cardiac workflow, Siemens) was used to analyse all CT images with multi-planar reconstructions and axial slices. In the applied workflow protocol (Cardiac) centrelines were automatically drawn in the right coronary artery (RCA), left main (LM), left anterior descending artery (LAD) and circumflex artery (Cx). The 17-segment model of the American Heart Association (AHA) was used to assess the coronary arteries [19]. An experienced researcher trained for objective assessment (NGE) performed the objective image quality measurements. Intravascular attenuation in HU was measured by manually drawing intravascular circular regions of interest (ROIs), image noise was defined as the standard deviation (SD) of the intravascular attenuation. The ROIs where drawn as large as possible while avoiding arterial walls and intravascular plaques. Epicardial fat attenuation (HU) was measured by drawing an ROI in the epicardial fat. CNR was calculated by intravascular attenuation minus epicardial fat attenuation, divided by the SD of the epicardial fat attenuation. Signal-to-noise ratio (SNR) was calculated by intravascular attenuation divided by the SD of the intravascular attenuation. Overall (of all coronary arteries) and segmental image quality for all 17 segments was assessed with help of intravascular attenuation, image noise, CNR and SNR. Literature shows a wide range of intravascular attenuation thresholds of 250–325 HU being used [20–23]. Since no consensus on the optimal enhancement exists, a threshold of 300 HU has been arbitrarily chosen. Furthermore, a CNR > 10 was considered diagnostic [24].

Both overall and segmental subjective image quality were assessed by an experienced researcher trained for subjective assessment (NGE) by using the 4-point Likert scale: 1 = poor, non-diagnostic image quality: major artefacts and/or low attenuation; 2 = sufficient image quality: minor artefacts and/or insufficient attenuation, however still diagnostic; 3 = good image quality: minimal artefacts and sufficient attenuation; 4 = excellent image quality: no artefacts and sufficient attenuation.

### Statistical analysis

Statistical analysis of the data was performed using the Statistical Package for Social Sciences (SPSS version 23.0, Chicago, Illinois). Categorical variables were expressed as absolute numbers and percentages; continuous variables are expressed as mean ± SD. The data was tested for normal distribution. In case of normally distributed data, the independent samples T-test was used to compare means between the different scan protocols. One-way ANOVA was used to compare means between groups. In case of a significant difference, a post hoc Tukey test or non-parametric Kruskal-Wallis test was used to compare means between different kV settings in the two scan protocols. For categorical variables, chi-square (χ^2^) was used to test differences between groups. Correlations were calculated using Spearmans’ correlation. All p*-*values were 2-sided and a p-value below 0.05 was considered statistically significant.

## Results

### Patient characteristics

Between July and October 2016, a total of 141 consecutive patients were eligible for inclusion. A total of 114 patients were included in the statistical analysis (48.2% men). A total of 27 patients were excluded from further statistical analysis because group sizes were too small; 3 patients scanned at 100 kV, 9 patients at 120 kV and 15 patients scanned with a retrospective ECG-gated helical protocol. Of the 114 patients, no patient had a CABG in their case history, one patient underwent PCI in the RCA with stent placement. No significant difference was found in most patient characteristics between the two scan protocols, except for age (p = 0.040) and HR (p < 0.001) (**Table 1**).

**Table 1:** Patient characteristics, scan settings and preparation of overall population and for different scan protocols.

Characteristic (n = 114)	Overall	High pitch (n = 81)	AS (n = 33)	p-value
**Age (year)**	**57.7 ± 12.0**	**59.2 ± 11.4**	**54.1 ± 11.4**	**0.040†**
**Sex**				**0.227**
**Men (n)**	**55**	**42**	**13**	
**Women (n)**	**59**	**39**	**20**	
**Height (m)**	**1.71 ± 0.10**	**1.72 ± 0.10**	**1.70 ± 0.09**	**0.558**
**Body weight (kg)**	**77.5 ± 16.0**	**78.9 ± 16.6**	**74.0 ± 14.2**	**0.138**
**BMI (kg/m**^**2**^**)**	**26.3 ± 4.5**	**26.7 ± 4.6**	**25.4 ± 4.2**	**0.184**
**BV (L)**	**4.7 ± 0.9**	**4.8 ± 0.9**	**4.6 ± 0.8**	**0.160**
**Men (L)**	**5.4 ± 0.6**	**5.4 ± 0.6**	**5.2 ± 0.5**	**0.297**
**Women (L)**	**4.1 ± 0.6**	**4.2 ± 0.7**	**4.1 ± 0.6**	**0.881**
**HR (bpm)**	**64.1 ± 11.2**	**60.9 ± 10.3**	**72.6 ± 9.1**	**<0.001†**
**Indication scan (n)**				**0.126**
**CAD**	**65**	**43**	**22**	
**Atypical chest pain**	**30**	**24**	**6**	
**Atrial fibrillation**	**8**	**8**	**-**	
**Other**	**11**	**6**	**5**	
**kV setting**				**0.017°**
**70 kV (n)**	**60**	**36**	**24**	
**80 kV (n)**	**37**	**32**	**5**	
**90 kV (n)**	**17**	**13**	**4**	
**Pharmacological preparation**				
**Oral beta blockade (n)**	**26**	**17**	**9**	**0.936**
**I.v. beta blockade (n)**	**18**	**9**	**9**	**0.448**
**Sublingual nitro-glycerine (n)**	**114**	**81**	**33**	

Note–Values are presented as means ± standard deviation or numbers.

High pitch = prospective ‘high pitch’ scan protocol; AS = prospective ‘adaptive sequence’ scan protocol; BMI = body mass index; BV = blood volume; HR = heart rate; CAD = coronary artery disease; I.v. = intravenous.

†Independent samples T-test showed a significant difference between the two groups.

°Chi-square test showed a significant difference in kV setting between the two groups.

### Scan protocol

In 81 (71.1%) scans, a high pitch protocol was used; an AS protocol was used in 33 (28.9%) scans. ATVS selected 70 kV in 60 (52.6%), 80 kV in 37 (32.5%), and 90 kV in 17 (14.9%) patients (**Table 1**). **Table 2** shows all scan parameters for the different scan protocols. Except for mAs_eff_ (p = 0.237) in the AS protocol, significant differences were found in all scan parameters (mAs_eff_, CTDI_vol_, DLP, and effective dose; p ≤ 0.002) between the different kV settings.

**Table 2:** Dose report and comparison for different kV settings.

Scan parameter					
High pitch (n = 81)	Overall	70 kV	80 kV	90 kV	p-value
**Effective tube current (mAs)**	505 ± 91	462 ±98	539 ± 67	539 ± 79	0.001†
**CTDI**_**vol**_ **(mGy)**	2.2 ± 1.0	1.3 ± 0.3	2.5 ± 0.3	3.8 ± 0.6	<0.001†
DLP (mGy*cm)	72 ± 24	51 ± 8	80 ± 8	111 ± 16	<0.001†
**Effective dose (mSv)**	0.7 ± 0.3	0.5 ± 0.1	0.8 ± 0.2	1.1 ± 0.2	<0.001†
**AS (n = 33)**					
**Effective tube current (mAs)**	347 ± 71	336 ± 79	396 ± 26	346 ± 15	0.237
**CTDI**_**vol**_ **(mGy)**	10.6 ± 5.1	8.2 ± 2.4	14.3 ± 2.6	20.9 ± 3.4	<0.001†
DLP (mGy*cm)	171 ± 75	135 ± 33	238 ± 53	309 ± 63	<0.001†
**Effective dose (mSv)**	3.3 ± 1.9	2.5 ± 0.9	5.1 ± 2.7	5.8 ± 2.1	0.002†

Note–Values are presented as means ± standard deviation.

High pitch = prospective ‘high pitch’ scan protocol; AS = prospective ‘adaptive sequence’ scan protocol; CTDI = CT dose index; DLP = dose length product.

^†^Kruskal-Wallis test showed significance between kV settings.

### Injection protocol

The injection protocols for men and women for different kV settings and different scan protocols are shown in **Table 3**. For men, main bolus volume ranged between 21.8–57.1 mL for high pitch and 33.9–62.9 mL for AS; mean flow rates ranged between 2.6–6.7 mL/s for high pitch and 3.3–6.0 mL/s for AS; IDR ranged between 0.8–2.0 g I/s for high pitch and 1.0–1.8 g I/s for AS; TIL ranged between 6.5–17.1 g I for high pitch and 10.2–18.9 g I for AS. For women, the main bolus volume ranged between 19.8–45.9 mL for high pitch and 27.8–61.7 mL for AS; mean flow rates ranged between 2.4–5.4 mL/s for high pitch and 2.7–5.8 mL/s for AS; IDR ranged between 0.7–1.6 g I/s for high pitch and 0.8–1.7 g I/s for AS; TIL ranged between 5.9–9.9 g I for high pitch and 8.3–18.5 g I for AS. No adverse events occurred during this study.

**Table 3:** Injection parameters (mean [ranges]) for both scan protocols for different kV settings.

High pitch (n = 81)				
Injection parameter	Sex	70 kV	80 kV	90 kV
**Flow rate (mL/s)**	**F**	3.0 [2.4–4.0]	4.1 [3.2–5.2]	4.9 [4.3–5.4]
	**M**	3.7 [2.6–4.4]	4.8 [4.1–5.5]	5.8 [5.2–6.7]
**Test bolus (mL)**	**F**	6.2 [4.9–8.0]	8.7 [6.9–11.1]	10.5 [9.0–12.0]
	**M**	7.8 [4.9–8.9]	10.2 [9.0–12.0]	12.6 [10.8–14.1]
**Main bolus (mL)**	**F**	25.1 [19.8–32.9]	34.8 [26.9–45.0]	41.5 [37.1–45.9]
	**M**	31.4 [21.8–37.8]	41.0 [34.9–48.0]	50.7 [46.0–57.1]
**TIL (g)**	**F**	7.5 [5.9–9.9]	10.4 [8.1–13.5]	12.5 [11.1–13.8]
	**M**	9.4 [6.5–11.3]	12.3 [10.5–14.4]	15.2 [13.8–17.1]
**IDR (g I/s)**	**F**	0.9 [0.7–1.2]	1.2 [1.0–1.6]	1.5 [1.3–1.6]
	**M**	1.1 [0.8–1.3]	1.4 [1.2–1.7]	1.7 [1.6–2.0]
**IT**	**F**	8 [8–9]	9 [8–9]	9 [9–9]
	**M**	8 [8–9]	9 [8–9]	9 [9–9]
**AS (n = 33)**				
**Injection parameter**				
**Flow rate (mL/s)**	**F**	3.2 [2.7–3.8]	3.9 [3.5–4.2]	5.2 [4.5–5.8]
	**M**	3.9 [3.3–4.5]	4.7 [4.0–5.4]	5.8 [5.5–6.0]
**Test bolus (mL)**	**F**	6.5 [5.0–8.0]	7.8 [6.8–8.8]	10.4 [9.0–11.8]
	**M**	8.5 [6.9–10.1]	9.9 [8.0–12.0]	12.4 [11.9–12.9]
**Main bolus (mL)**	**F**	32.8 [27.8–40.1]	39.8 [35.8–43.8]	54.3 [46.9–61.7]
	**M**	41.3 [33.9–48.1]	49.9 [41.7–58.0]	61.0 [59.1–62.9]
**TIL (g)**	**F**	9.9 [8.3–12.0]	11.9 [10.7–13.1]	16.3 [14.1–18.5]
	**M**	12.4 [10.2–14.4]	15.0 [12.5–17.4]	18.3 [17.7–18.9]
**IDR (g I/s)**	**F**	1.0 [0.8–1.1]	1.2 [1.1–1.3]	1.6 [1.4–1.7]
	**M**	1.2 [1.0–1.4]	1.4 [1.2–1.6]	1.7 [1.7–1.8]
**IT**	**F**	10 [10–11]	10 [10–10]	11 [10–11]
	**M**	11 [10–11]	11 [10–10]	11 [11–11]

Note–Values are presented as means [range]

High pitch = prospective ‘high pitch’ scan protocol; AS = prospective ‘adaptive sequence’ scan protocol; TIL = total iodine load; IDR = iodine delivery rate; IT = injection time

### Image quality

#### Overall objective image quality

**Fig 2:**
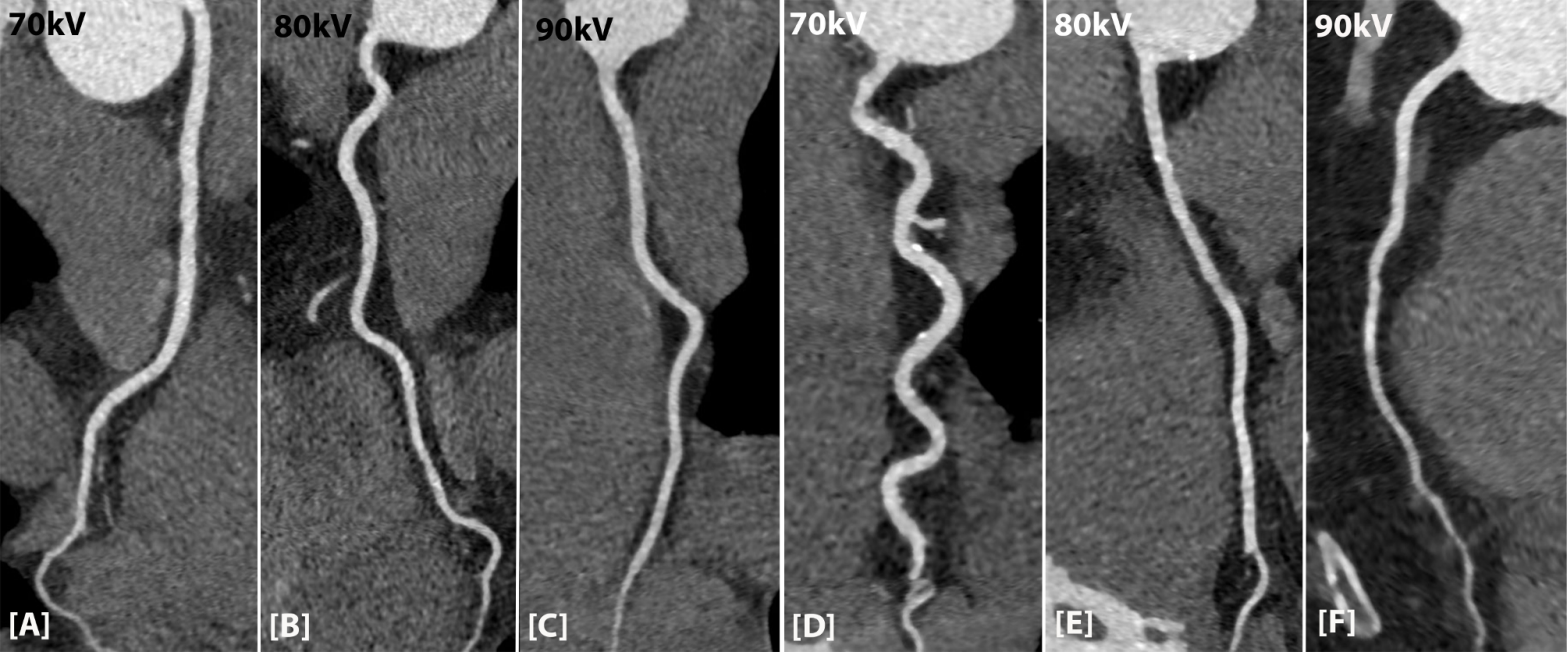
Attenuation profile in the RCA for the high pitch and AS protocol and different kV settings. [A-C] Attenuation in the RCA of the high pitch protocol for the three different kV settings. [D-F] Attenuation in the RCA for the AS protocol for the three different kV settings.

Overall mean attenuation of all coronary arteries was diagnostic in 98.2% of all scans (112/114 patients). Mean attenuation for kV settings was 378 ± 48 HU (70 kV), 358 ± 37 HU (80 kV), 360 ± 38 HU (90 kV) for the high pitch protocol and 354 ± 33 HU (70 kV), 375 ± 52 HU (80 kV) and 336 ± 44 HU (90 kV) for the AS protocol (**Table 4**). Mean attenuation for men was 369 ± 40 HU for high pitch and 374 ± 42 HU for AS. For women, this was 365 ± 46 HU for high pitch and 343 ± 28 HU for AS. Mean overall CNR was 14 ± 4 (70 kV), 13 ± 3 (80 kV) and 14 ± 4 (90 kV) for the high pitch protocol and 16 ± 4 (70 kV), 17 ± 4 (80 kV) and 18 ± 2 (90 kV) for the AS protocol. No significant difference was found in parameters between the kV settings. When comparing attenuation and CNR levels between men and women for both scan protocols at different kV settings, no significant difference was found. **Fig 2** shows the attenuation profile in the RCA for the two scan protocols (high pitch A-C and AS D-F) and different kV settings.

**Table 4:** Overall objective image quality for every scan protocol and kV setting.

Scan parameter					p-value
High pitch (n = 81)	Overall	70 kV	80 kV	90 kV	
**Coronary attenuation (HU)**	367 ± 43	378 ± 48	358 ± 37	360 ± 38	0.141
**Image noise**	43 ± 6	43 ± 7	42 ± 6	45 ± 7	0.256
**CNR**	14 ± 4	14 ± 4	13 ± 3	14 ± 4	0.602
**SNR**	9 ± 2	10 ± 2	9 ± 1	9 ± 2	0.353
**AS (n = 33)**					
**Coronary attenuation (HU)**	355 ± 37	354 ± 33	375 ± 52	336 ± 44	0.296
**Image noise**	40 ± 7	40 ± 6	38 ± 12	38 ± 6	0.676
**CNR**	16 ± 4	16 ± 4	17 ± 4	18 ± 2	0.635
**SNR**	10 ± 2	10 ± 2	11 ± 3	10 ± 2	0.261

Note–Values are presented as means ± standard deviation.

High pitch = prospective ‘high pitch’ scan protocol; AS = prospective ‘adaptive sequence’ scan protocol; SD = standard deviation; CNR = contrast-to-noise ratio; SNR = signal-to-noise ratio.

**Fig 3:**
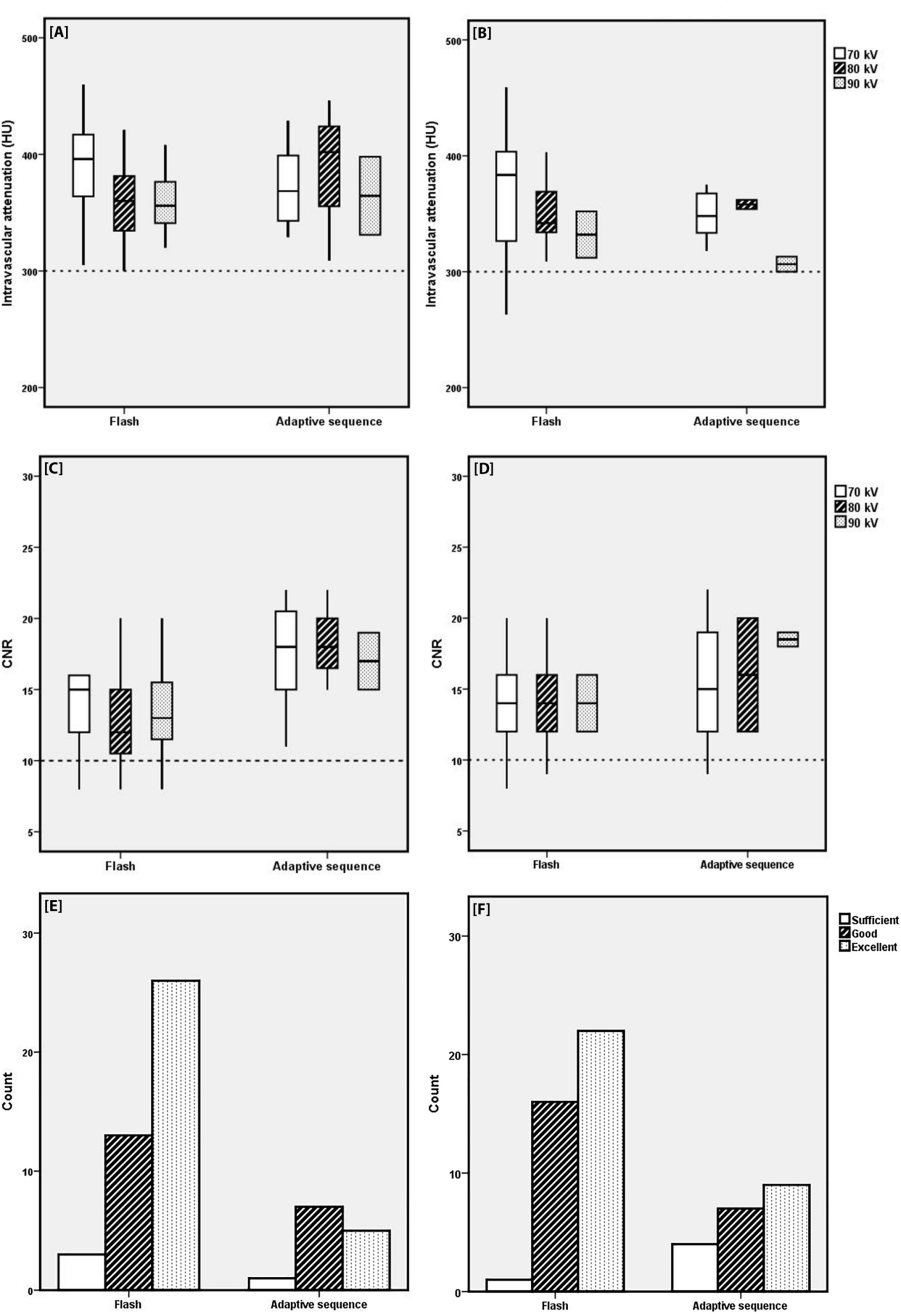
**Attenuation, CNR levels and Likert scale for different scan protocols and different kV settings for men [A-C-E] and women [B-D-F].** *[A+B] The overall attenuation was > 300 HU*, *except for two scans in the high pitch group scanned at 70 kV for women [B]*. *[C+D] CNR levels were below 10 in 11 scans; 4 scans in both the 70 kV and 80 kV group*, *1 scan in the 90 kV group for the high pitch protocol and 2 scans in the 70 kV group for the AS protocol*. *[E+F] The Likert scales and counts assigned to the two scan protocols*. *No scan was* graded as ‘poor’.

**Fig 3** shows the overall attenuation, overall CNR and Likert scores for different scan protocols and different kV settings for both men and women. Mean attenuations (**Fig 3A and 3B**) for different kV settings were diagnostic (> 300 HU), except for the scans of two women in the high-pitch group at 70 kV. One scan was regarded as diagnostic (263 HU), the other as non-diagnostic (264 HU). However, no extra scan was made, since the clinical query could be answered.

CNR levels were below 10 in 11 scans (**Fig 3C and 3D**); 4 scans in both the 70 kV and 80 kV groups, 1 scan in the 90 kV group for the high pitch protocol, and 2 scans in the 70 kV group for the AS protocol. All these scans were considered diagnostic with regard to subjective image quality.

**Fig 4:**
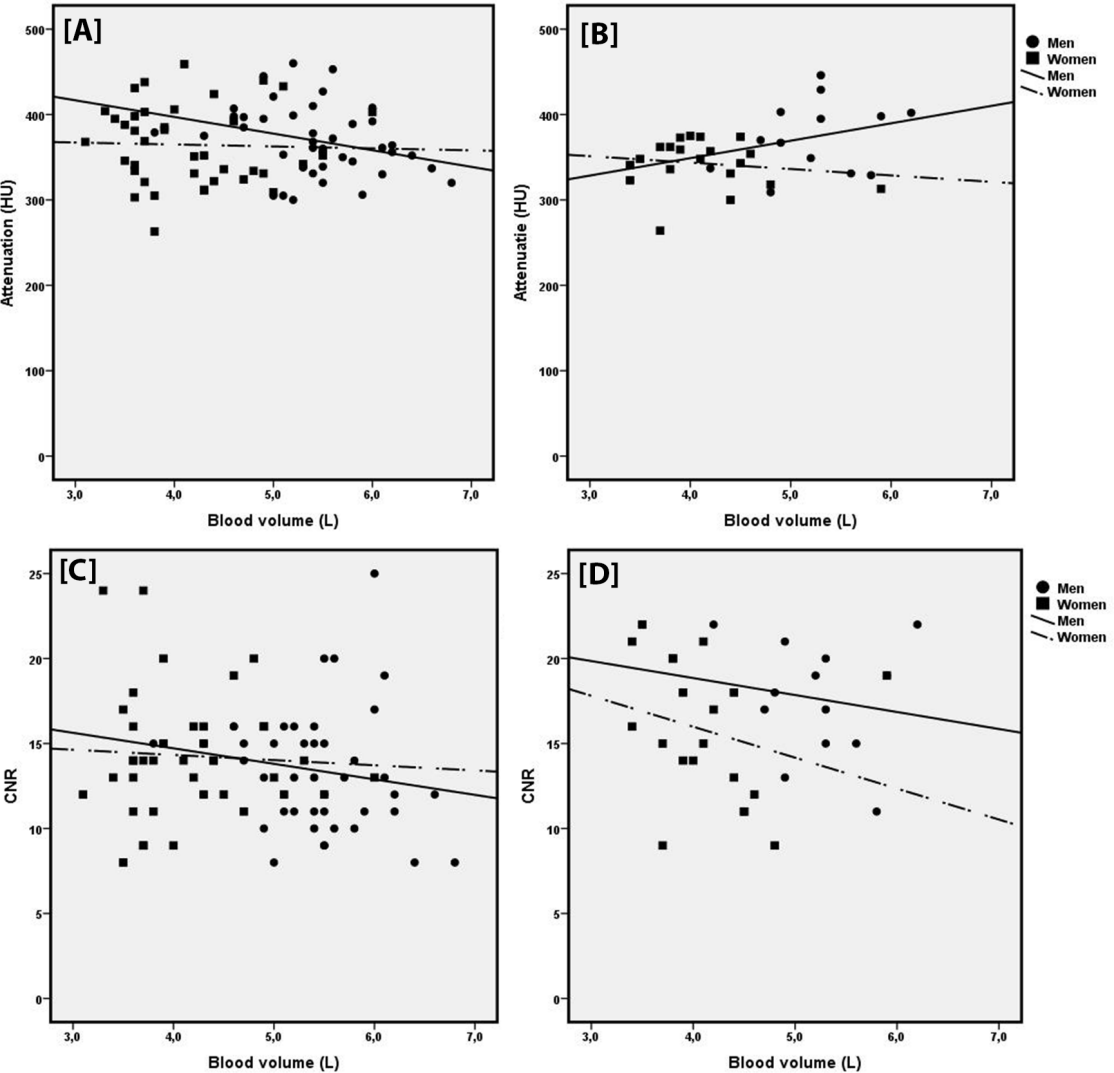
**Correlation between attenuation and blood volume for men and women for the high pitch and AS protocol [A+B] and correlation between CNR and blood volume for men and women and the high pitch and AS protocol [C+D].** *Correlation between attenuation and blood volume was slightly negative in men and women for the high pitch protocol (r = -0*.*296 and r = -0*.*033) and women for the AS protocol (r = -0*.*153)*. *Men scanned with the AS protocol showed a slight positive correlation between HU and blood volume (r = 0*.*268) [A+B]*. *Correlation between CNR and blood volume was slightly negative in men for the high pitch protocol (r = -0*.*213) and no correlation was found between CNR and blood volume in women for the high pitch protocol*. *For the AS protocol*, *for both men and women a slight negative correlation was found (r = -0*.*155 and r = -0*.*258) [C+D]*. *All correlations showed no significance (p > 0*.*05)*.

Correlation between BV and HU for men and women and the two scan protocols showed a negative trend between HU and BV (**Fig 4**), except for men scanned with the high pitch protocol. No correlation was significant. Correlation between BV and CNR showed slight negative trend for men scanned with the high pitch and AS protocol, as for women scanned with the AS protocol, although no correlation was significant. CNR and BV showed no correlation for women scanned with the high pitch protocol.

#### Segmental objective image quality

A total of 1.661 segments were present; 1.585 (95.4%) of these segments were assessable. A total of 4.6% of segments were not assessable due to bolus timing, movement artefacts or small arteries. The segments not assessable most often were the distal segments: segment 4 of the RCA (6.1% of 114 scans), segments 9 and 10 of the LAD (10.5% and 7.9%) and segments 12, 13 and 14 of the Cx (13.2%, 4.4% and 13.2%). Mean attenuation was ≥ 300 HU in 88.7% of all segments. Segmental attenuation was < 300 HU due to multiple potential causes such as bolus timing, movement artefacts and/or small arteries. A low attenuation was mostly seen in the distal segments: intravascular attenuation was below 300 HU in only 8 proximal segments (segments 1, 5, 6 or 11; 0.5% of all segments: 1 for men and 7 for women). However, although the attenuation was below 300 HU in some coronaries, these could still be (adequately) assessed for the presence of plaques by the cardiac radiologist.

CNR was below 10 in 10.8% of all segments, 12.9% in the high pitch protocol and 5.6% in the AS protocol. A CNR < 10 was mostly seen in the LAD side branches (segment 9 and 10), the distal Cx and side branch (segment 14 and 15) and the ramus intermedius (segment 17).

#### Overall and segmental subjective image quality

No scan was overall graded with a non-diagnostic overall subjective image quality. For high pitch, 4 scans were graded with Likert score 2 (4.9%), 29 scans with Likert score 3 (35.8%) and 48 scans with Likert score 4 (59.3%). The AS protocol resulted in 5 scans with Likert score 2 (15.2%), 14 scans with Likert score 3 (42.4%) and 14 scans with Likert score 4 (42.4%). No significant difference in subjective image quality between the high pitch and AS protocol (p = 0.366) was found. Furthermore, no significant difference in subjective image quality between men and women was found for the high pitch protocol (p = 0.828) or AS protocol (p = 0.667).

**Fig 5:**
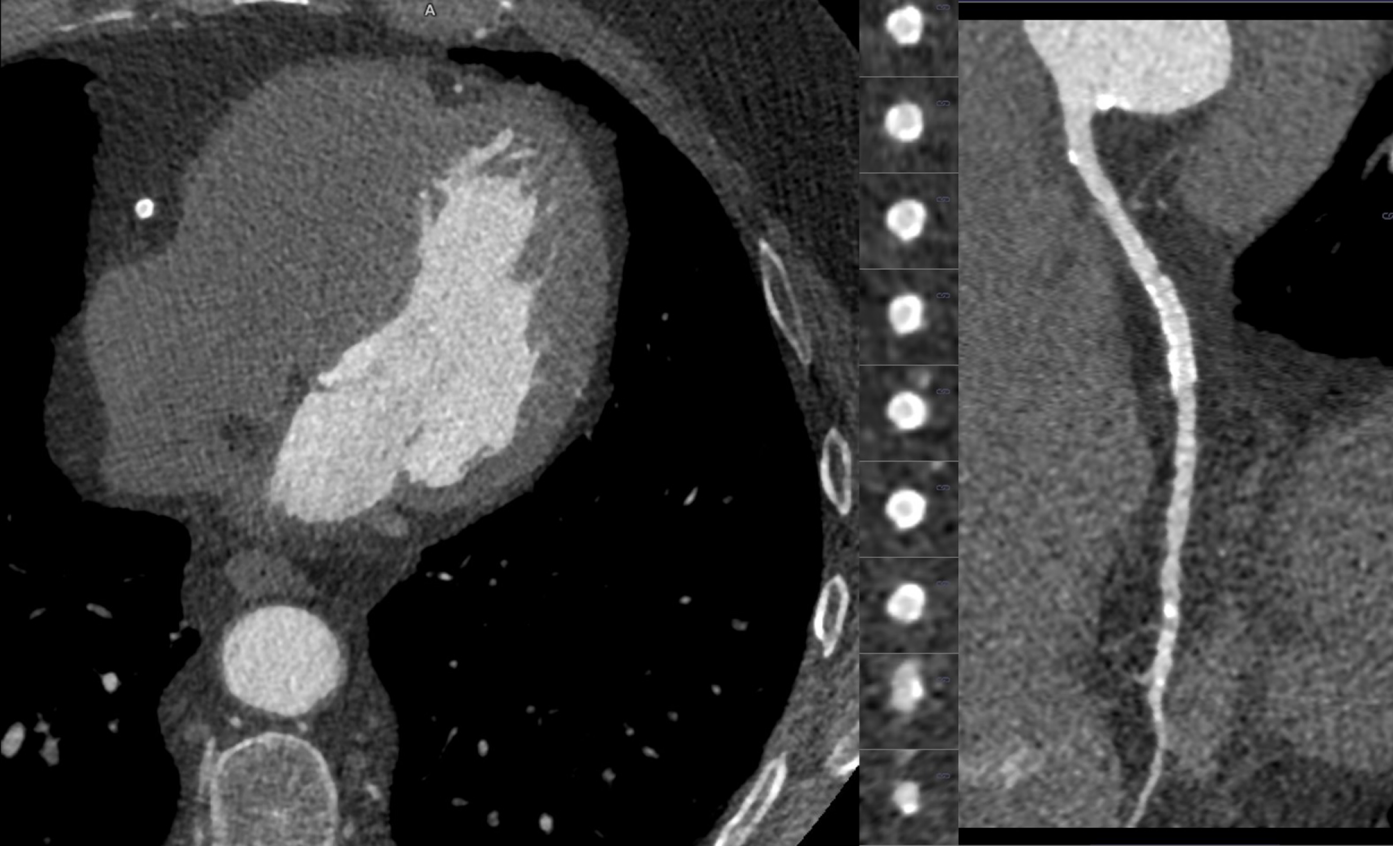
Patient with a stent implantation in segment 2 of the RCA. Patient with a stent placement in segment 2 of the RCA. Scanned with a high pitch protocol and 90 kV. Image quality was graded as excellent. The centreline RCA and axial slices are visible.

A Likert score of 4 was applicable in 44.9% of segments (47.2% high pitch; 7.5% AS), Likert score 3 in 34.0% (33.8% high pitch; 34.4% AS) and Likert score 2 in 16.4% (15.4% high pitch; 18.9% AS) of segments. In all above-mentioned non-assessable segments, subjective image quality was scored as 1 (4.6%). This was mostly seen in the distal and side branch segments. Segments 12 (1^st^ obtuse marginal Cx) and 14 (2^nd^ obtuse marginal Cx) for the high pitch and segment 10 (2^nd^ diagonal LAD) and 14 for the AS protocol were scored as 1 most frequently. In only 5 segments (0.3% of all segments), 2 in high pitch and 3 in AS, subjective image quality was scored as 1 in the proximal segments due to movement artefacts (3 scans) and a small Cx artery (2 scans). Other reasons for a non-diagnostic subjective image quality were low attenuation, movement artefacts and small arteries. One patient (scanned with a high pitch protocol and 90 kV) had a stent in the RCA (segment 2), this segment was assessable and graded as excellent image quality (**Fig 5**).

## Discussion

The use of personalised injection protocols adapted to patient’s BV and ATVS resulted in diagnostic overall intravascular attenuation in 98.2% scans, and in a CNR ≥ 10 in 90.4% scans (**Fig 3A–3D**). Segmental intravascular attenuation was ≥ 300 HU in 71.5% of all segments and in 85.7% of proximal segments. Overall diagnostic subjective image quality was reached in all scans and in 95.4% of coronary segments.

The CNR levels (**Fig 3C and 3D**) showed no significant difference between men and women. Thus, with a lower amount of CM volume (the smallest volume injected in this study was 19.8 mL), a similar HU and CNR level can be reached in the different kV settings for both men and women. When testing for a correlation between BV and HU and BV and CNR, almost all correlations showed a slightly negative trend. Surprisingly, the correlation between HU and blood volume showed a slight positive trend for men in the AS protocol. The small group sizes have to be taken into account here (13 men in the AS group). With such small group sizes, small differences alter a slope. However, not any of the correlations was significant, and the positive trend can also be seen as a coincidence.

**Fig 3A and 3B** also show that the overall intravascular attenuation of different scan protocols was diagnostic, except for two scans in the high pitch and AS groups (both 70 kV). Image quality in CCTA scanning is susceptible to multiple factors. Intravascular attenuation, motion artefacts, vessel diameter and scan parameters are all factors that could influence the image and diagnostic quality of a scan. The 300 HU threshold was no guarantee for an assessable coronary artery, illustrating that setting a definite attenuation threshold for (non-)diagnostic image quality is difficult and up for discussion.

The scanner and test bolus would ideally correct for HR to prevent late scan timing. Unfortunately, irregular HR during the scanning process can still result in a suboptimal image quality due ‘missed’ peak attenuation (scanning either too late or too early). It is known that with a decreased CO the intravascular attenuation peak is delayed due to decreased circulation of CM [15]. Perhaps the correction for CO in the formula was not specific enough, and a different measurement or calculation of CO is needed (e.g. non-invasive finger-cuff measurement).

The individualisation of CM injection protocols is gaining more interest in the overall transition to personalised medicine. Especially in patients with cardiovascular diseases, the concern of development of CIN still exists. Although the relation between CIN and CTA has been called into question [25], personalisation of CM injection protocols is still desirable since low CM volumes will decrease incidence and risk of CIN. Many techniques have been studied to adapt CM injection protocols to the individual patient, e.g. adaptation to tube voltage and BW [11–13, 26–28]. In the current study, the use of ATVS shifted tube voltage settings to low kV settings (70, 80 and 90 kV). A tube voltage of 100 kV was never selected by the software, and only 3 and 9 patients were scanned with 110 kV and 120 kV, respectively. Both tube voltage and body weight adapting techniques previously helped to significantly reduce CM volume in patients undergoing CCTA. Between these two techniques, the adaptation of CM injection protocols to tube voltage is the most significant in terms of CM reduction.

The current study assessed the feasibility of further adaptation (besides tube voltage) of CM injection protocols to BV. The advantage of adapting CM protocols to BV is that the estimated BV in litres is calculated differently for men and women. Previously, the CM injection protocol was adapted to BW categories in our department (e.g. <60 kg, 60–70 kg). With that method, no distinction between sex or kV setting was made. The intravascular attenuation for different weight groups between men and women showed a wider standard deviation for women, with attenuation values of up to 900 HU. This shows that some women received a CM bolus that was too high for their body habitus. Moreover, borderline patients (patients of e.g. 61 kg or 69 kg) would also receive either too much or too little CM volume. When adapting for BV, a distinction between sex is made and a more continuous measurement is used, therefore this problem should not occur. A continuous adaptation scale of the injection protocols to BW could also be feasible, however a correction for sex of the patient will not be made. Therefore, BV based CM volumes can be considered more patient specific compared to BW. Lastly, the formula used was implemented in a locked Excel file and could easily be adapted and implemented in daily clinical practice for other scanners in other hospitals (**Fig 1**).

This study has some limitations; no direct comparison between the current method and BW adapted protocols could be made, since the BW adapted protocols did not take tube voltage into account. However, this study was a feasibility study and did not focus on a direct comparison. A second limitation is that the sample sizes in the higher kV levels and helical scan protocol were so small, that statistical analysis was not possible. It would be interesting to see if the formula works in the higher kV levels and helical scan protocol as well.

To conclude, adaptation of CM injection protocols to the patient’s BV and ATVS in CCTA results in an overall and segmental diagnostic image quality in almost all scans. It is easy to use, making this a promising and feasible technique.
